# Lightweight Modification of Polypropylene Cable Insulation Materials Doped with Hollow Glass Microspheres

**DOI:** 10.3390/polym17243321

**Published:** 2025-12-16

**Authors:** Xindong Zhao, Dongxu Luo, Kai Wang, Jiaming Yang, Ling Weng, Xiongjun Liu, Xiao Han, Xin Yao

**Affiliations:** 1 State Key Laboratory of High-Efficiency Special Cable Technology, Harbin University of Science and Technology, Harbin 150080, China; 2 Jiangsu Shangshang Cable Group Co., Ltd., Liyang 213300, China

**Keywords:** overhead cables, lightweighting, polypropylene, hollow glass microspheres

## Abstract

Overhead transmission lines have long relied on cross-linked polyethylene (XLPE) insulation. The production of XLPE insulation requires silane cross-linking, which generates by-products, consumes high energy, and results in poor recyclability-retired XLPE insulation can only be disposed of through incineration or landfilling. Additionally, its high density leads to increased cable weight and sag, reducing the service life of the cables. Therefore, there is an urgent need to develop recyclable and lightweight insulation materials. In this study, recyclable polypropylene (PP) was used as a substitute for XLPE. Hollow glass microspheres (HGM) were incorporated to reduce weight, and hydrogenated styrene-ethylene-butylene-styrene block copolymer (SEBS) was added for toughening, thereby constructing a PP/HGM/SEBS ternary composite system. The results show that the introduction of HGM into the PP matrix effectively reduces the material density, decreasing from 0.890 g/cm^3^ (pure PP) to 0.757 g/cm^3^—a reduction of 15%. With the addition of SEBS, the mechanical properties of the composite are significantly improved: the tensile strength increases from 14.94 MPa (PP/HGM) to 32.40 MPa, and the elongation at break jumps sharply from 72.02% to 671.22%, achieving the synergistic optimization of “weight reduction” and “strengthening-toughening”. Electrical performance tests indicate that the PP/HGM/SEBS composite exhibits a volume resistivity of 1.66 × 10^12^ Ω·m, a characteristic breakdown strength of 108.6 kV/mm, a low dielectric loss tangent of 2.76 × 10^−4^, and a dielectric constant of 2.24. It achieves density reduction while maintaining low dielectric loss and high insulation strength, verifying its feasibility for application in lightweight insulation scenarios of overhead transmission lines.

## 1. Introduction

**Figure 1:**
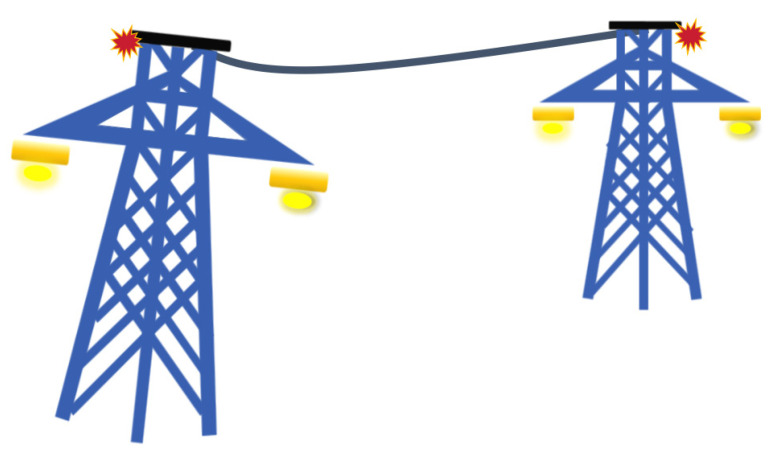
Low sag phenomenon caused by the excessive weight of the cable.

Overhead cables have become an integral part of urban and rural power grids, offering advantages such as no need for excavation, rapid installation, low investment costs, and convenient maintenance. They are particularly suitable for long-distance and large-span power transmission scenarios. To ensure the safe and reliable operation of transmission lines, their insulation materials must simultaneously possess excellent heat resistance, dielectric properties, and mechanical strength [1,2,3,4,5]. Cross-linked polyethylene (XLPE) has been the mainstream insulation choice for high-voltage overhead cables since the 1970s due to its high heat resistance grade and high breakdown strength [6,7]. However, the cross-linking process of XLPE is accompanied by by-products such as methane and requires vulcanization under high-temperature and high-pressure conditions, resulting in high energy consumption and long production equipment requirements. The three-dimensional thermosetting network formed after cross-linking prevents retired cables from being remelted and granulated for recycling; they can only be disposed of through incineration or landfilling. This not only wastes petrochemical resources but also emits carbon dioxide and toxic flue gases, which is contrary to the development direction of the circular economy under the “dual carbon” goal [8,9,10]. In addition, XLPE has a high density of up to 0.93 g/cm^3^, leading to excessive insulation weight of 10 kV cables over a kilometer scale. This causes a simultaneous increase in sag and tower load (as shown in Figure 1), becoming a technical bottleneck in long-span and large-crossing projects. Therefore, the development of new lightweight thermoplastic recyclable insulation materials has become a top priority for the green upgrading of overhead cables [11,12].

Polypropylene (PP) features a main chain composed of carbon-carbon bonds without polar side groups, exhibiting a volume resistivity comparable to that of XLPE and a continuous operating temperature range extendable to 90–100 °C. It can be extruded without the need for peroxide cross-linking, and retired PP-based cables can be remelted and granulated to achieve a closed-loop cycle of “insulation-recycling-re-insulation”, making it the most promising alternative matrix [13,14]. However, the high crystallinity of PP leads to coarse spherulites at room temperature and low notched impact strength, resulting in brittleness and cracking risks during cable installation, low-temperature operation, and joint assembly. Additionally, its high modulus increases construction difficulty and thermomechanical stress concentration. Therefore, toughening modification is indispensable to meet the mechanical reliability requirements of overhead cables throughout their service life [15,16]. Melt blending modification, which introduces a flexible phase while retaining PP’s thermoplasticity, is the preferred approach for balancing toughening effects and recyclability. To address PP’s poor impact strength, Gao Mingze et al. utilized styrene-ethylene-butylene-styrene block copolymer (SEBS) as a toughening filler and incorporated nano-silica (nano-SiO_2_) to enhance the electrical properties of the nanocomposite [17]. Nam et al. prepared PP blends via melt mixing with thermoplastic elastomer SEBS, enabling recyclable PP/SEBS composites to achieve performance comparable to XLPE and realize sustainable high-voltage insulation [18]. Although modified PP can solve the problems of cable recyclability and insufficient toughness when replacing XLPE, its intrinsic density is similar to that of XLPE, failing to meet the lightweight requirement for long-span applications. Thus, lightweight modification of PP is imperative [19,20].

Hollow glass microspheres (HGM) have a true density of only 0.2–0.4 g/cm^3^ [21,22], and have been proven to achieve rapid weight reduction of over 10% at low filling contents. Zhaolin Zhu et al. studied composites fabricated from HGM and polypropylene (PP), with the minimum density reaching 0.85 g/cm^3^ [23]. Francesco Galvagnini et al. developed novel paraffin-containing PP-based syntactic foams; the introduction of HGM into PP effectively reduced the foam density [24]. However, blending PP with HGM, while reducing density, inevitably leads to decreased mechanical properties, which deteriorate significantly with increasing HGM content. Ren et al.’s experiments demonstrated that when the HGM dosage exceeds 15 phr, interfacial debonding between microspheres and the matrix causes a sharp decline in tensile and impact properties, resulting in a prominent “weight reduction-brittleness” contradiction [25]. Zhu Z et al.’s research also confirmed that the tensile strength and impact strength of the composites decrease with increasing HGM content [23]. This is attributed to the tendency of HGM to agglomerate and poor dispersion in the PP matrix, necessitating improvements in HGM dispersion. To maintain mechanical strength, Baptista et al. employed PP-g-MAH (maleic anhydride-grafted polypropylene) as a compatibilizer to enhance the adhesion between the polymer matrix and microspheres [26].

Existing studies on the “weight reduction and high strength” of HGM/PP composites have mostly focused on industries such as automotive and aerospace. However, systematic evaluation of their electrical insulation properties (including breakdown strength, dielectric loss, and volume resistivity) is lacking. Based on this research gap, this study for the first time introduces HGM into the field of PP-insulated cables to mitigate issues of excessive sag and tower load. To address the mechanical performance shortcomings, the elastomer SEBS is incorporated as a compatibilizer and toughening agent, constructing a PP/HGM/SEBS ternary blend system. Systematic evaluations are conducted on its density, mechanical properties, dielectric properties, and breakdown characteristics, aiming to provide data support and theoretical basis for the engineering application of polypropylene-based insulation materials and promote the industrialization of recyclable and lightweight overhead cables.

## 2. Material Preparation and Experimental Methods

### 2.1. Material Preparation

The samples were prepared using polypropylene pellets (grade T30S, manufactured by Sinopec (Beijing, China)) as the base material, with hollow glass microspheres (Grade HL42, manufactured by Zhengzhou Saintlite (Zhengzhou, China)). as the filler and thermoplastic elastomer SEBS (manufactured by Kraton Corporation (Houston, TX, USA)). The materials were compounded via melt blending. For simplicity in subsequent descriptions, the material compositions and corresponding sample names are summarized in Table 1.

**Figure 2:**
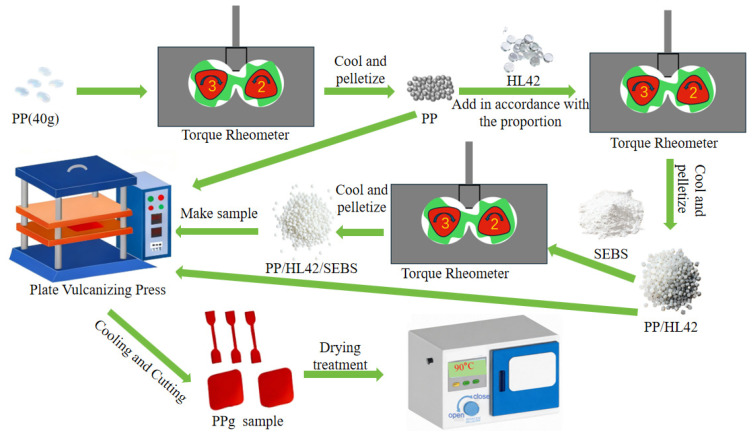
Process diagram of material preparation.

The specific process is as follows: the temperature of each zone of the torque rheometer was set at 180 °C. After the temperature was reached, the speed was set to 60 rpm/min, and the accurately weighed PP pellets were added. After mixing for about 6 min, the molten PP compound was obtained. When the torque curve became stable, the speed was adjusted to 30 rpm/min, and the accurately weighed hollow glass microspheres (HL42) were added. After mixing for 3 min and the torque curve became stable again, and the PP/HL42 pellets were obtained. On this basis, SEBS was added to obtain the PP/HL42/SEBS pellets. Step two: The three types of pellets were melted in a flat vulcanizing machine at 180 °C for 15 min. Then, the pressure increased, and the pellets were pressed for 5 min each at 5 MPa, 10 MPa, and 15 MPa. After that, the pellets were cooled in a mold with water for 5 min to form the lightweight polymer samples for characterization and testing. The process is shown in Figure 2.

### 2.2. Experimental Methods

The stress–strain test is a method for assessing material performance under axial tensile force until fracture. It provides a stress–strain curve to analyze key parameters like elongation at break, tensile strength, yield strength, and yield point. For the tensile test, specimens are made into dumbbell shapes 1 mm thick with a 50 mm gauge length and 20 mm initial grip separation. The testing machine stretches at 50 mm/min at room temperature, with five repetitions for each material. The test is conducted using an MTS CMT6000 electronic tensile tester (Manufactured by MTS Systems (China) Co., Ltd., Shanghai, China) in accordance with ISO 527-3:2018 [27].

Density Testing: The density of the composite materials was measured using a DH300 densimeter (Manufactured by Shenzhen Dahometer Instrument Co., Ltd., Shenzhen, China). Before testing, deionized water (resistivity ≥ 18.2 M Ω·cm, density 0.9982 g/cm^3^) was used as the medium, and its temperature was adjusted to 23 °C to avoiad interference from impurities and temperature fluctuations. Defect-free samples (diameter 5 mm, thickness 5 mm) were cut, with three replicates prepared for each group. After wiping the surface with anhydrous ethanol, the samples were dried in a vacuum oven at 50 °C for 2 h and then cooled for later use. The instrument was first turned on and preheated for 30 min. The clean measuring basket was then immersed in water (ensuring no air bubbles were attached) and zero-point calibration was performed. During the formal test, the mass of the sample in air was measured first, followed by placing the sample in the basket and completely submerging it in water (avoiding air bubble generation) to measure its mass in water. The average value of three parallel tests was taken as the final data.

Shore Hardness Testing: D Shore hardness tester, model LX-D (mechanical type) from Shanghai Yizong Precision Instrument Co., Ltd., Shanghai, China was used. The sample was placed on a firm plane. The hardness tester was held such that the needle was at least 12 mm away from the edge of the sample. The presser foot was steadily pressed onto the sample without any vibration and kept parallel to the sample surface to ensure the needle penetrated the sample vertically. The applied force was sufficient to ensure tight contact between the presser foot and the sample. The reading was taken at the specified time, which is 15 s for thermoplastic rubber. The hardness was measured at five different locations on the sample, with each location at least 6 mm apart, and the average value was taken.

Melt Flow Rate (MFR) Testing: Melt flow rate (MFR) testing is primarily used to evaluate the processability and flow characteristics of materials. MFR refers to the mass of polymer melt that flows through a standard capillary within a certain time under specific temperature and pressure conditions (measured in g/10 min). In this study, a PXRZ-200C (Jilin Puxin Precision Instrument Equipment Co., Ltd., Changchun, China) melt flow rate tester was used to conduct MFR tests on the materials. The test was performed in accordance with ISO 1133-1:2011 [28], with the equipment parameters set at a temperature of 230 °C and a load of 2.16 kg. The mass flowing through the standard orifice was measured over a period of 10 min.

**Figure 3:**
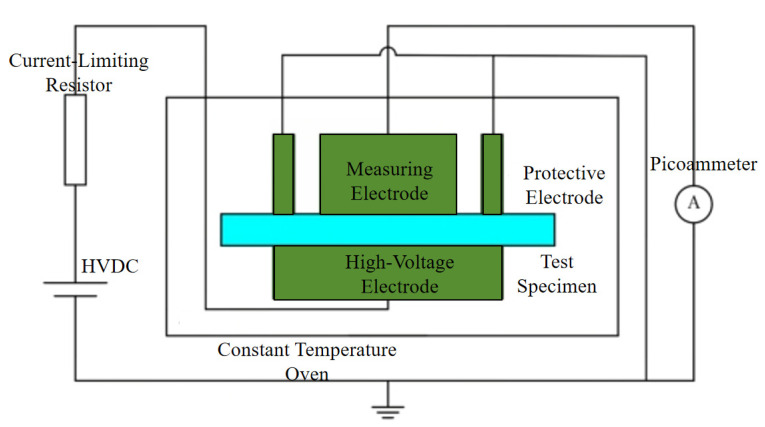
Schematic diagram of DC volume resistivity testing device.

Volume Resistivity Testing: In this study, a three-electrode high-resistance tester (model ZC-36 Shanghai Sixth Electric Meter Factory Co., Ltd., Shanghai, China) was used to characterize the direct current volume resistivity of the samples under ambient temperature conditions. As shown in Figure 3, the testing system mainly consists of a high-voltage DC power supply, a three-electrode device, an electrostatic detection unit, a current-limiting resistor, and a constant-temperature chamber. To ensure effective contact between the sample and the electrodes, an aluminum conductive layer was evenly applied to both sides of the sample before testing. During the test, a constant-temperature environment of 25 °C was maintained in the chamber, and a continuous DC electric field of 10 kV/mm was applied to the sample with a thickness of approximately 100 μm for 30 min. The steady-state resistance value was recorded, and the volume resistivity was calculated according to Equation (1). At least five parallel samples were tested for each material, and the arithmetic mean was taken as the final result after verifying the repeatability of the data.

(1) $$\rho_{V}=R_{V}\times \frac{\pi {\left(D_{1}+g\right)}^{2}}{4h}$$

where *ρ_V_* represents the volume resistivity, Ω·cm; *R_V_* denotes the volume resistance of the sample, Ω; *h* is the thickness of the sample, m; *D*_1_ is the diameter of the measuring electrode, with a value of 0.05 m; *g* represents the spacing between the measuring electrode and the guard electrode, with a value of 0.002 m.

AC Breakdown Test: This test characterizes the extreme ability of polymer materials to maintain their insulating properties under high AC electric fields. When the electric field strength reaches a critical value, the dielectric transitions from an insulating state to a conducting state. This critical electric field strength is known as the AC breakdown field strength, which is calculated using Equation (2):

(2) $$E=\frac{U}{d}$$

where *U* is the voltage at which the insulating material breaks down, kV; *d* is the thickness of the test sample, mm; *E* is the breakdown field strength of the sample, kV/mm.

The breakdown field strength is further analyzed using a two-parameter Weibull distribution to obtain the shape parameter and characteristic breakdown field strength. The probability density function of the Weibull mathematical model is given in Equation (3).

(3) $$f(x)=\left[\frac{b}{\theta -x_{0}}{\left(\frac{x-x_{0}}{\theta -x_{0}}\right)}^{b-1}\right]\{\exp\left[-{\left(\frac{x-x_{0}}{\theta -x_{0}}\right)}^{b}\right]\}$$

where *x*_0_ represents the minimum expectation; *θ* is called the scale parameter; *b* is the shape parameter, also known as the Weibull slope, which is an indicator of data dispersion.

When the insulating material is subjected to an external electric field *E* for a time *t*, and the failure probability is characterized using the Weibull distribution, it can be assumed that *x*_0_ is 0. The expression can then be simplified as shown in Equation (4).

(4) $$P(E)=1-\exp[-{\left(\frac{E}{E_{0}}\right)}^{\beta }]$$

where *P* represents the cumulative failure probability; *E* is the measured breakdown strength of the sample; *E*_0_ is the electric field strength when the cumulative breakdown probability of the sample reaches 63.28%. The dispersion of the sample’s breakdown strength is characterized by *β*, which is called the shape parameter.

**Figure 4:**
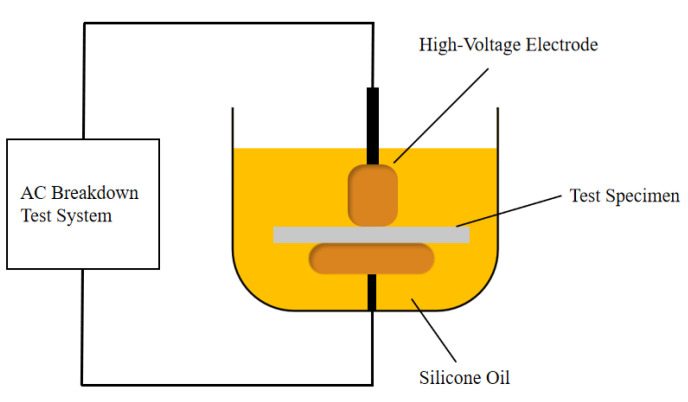
AC breakdown test setup.

The test was conducted in accordance with the standard IEC 60243-1:2013 [29], using a cylindrical electrode test system (The structure diagram is shown in Figure 4; the high-voltage electrode has a diameter of 35 mm, and the ground electrode has a diameter of 50 mm). The electrodes were immersed in dimethyl silicone oil to prevent surface flashover. During the test, the sample (thickness: 50 μm; radius: 4 cm) was laid flat on the surface of the ground electrode to ensure tight contact between the electrodes and the sample. Subsequently, the device was placed in a constant-temperature oil bath to measure the AC breakdown strength at 30 °C with a voltage rise rate of 0.5 kV/s. For each material composition, more than 10 repeated tests were performed at the specified temperature. The shape parameter and characteristic breakdown field strength were obtained through statistical analysis using the two-parameter Weibull distribution.

Dielectric Loss Testing: Before the experiment, the capacitance disc was set to the calibration value, and all balance knobs were set to zero. A 0.2 mm sample was placed in a three-electrode system, and a 200 V power frequency was applied to the high-voltage electrode. The capacitance ratio disc was first roughly and then finely adjusted, and the sensitivity was gradually increased until the pointer was zeroed. Then, the tan δ potentiometer was adjusted to zero the meter. The values of *C_x_* and tan *δ* were directly read. The relative permittivity *ε_r_* is the ratio of the capacitance with the dielectric material *C_x_* to the capacitance in vacuum *C*_0_ under the same electrode configuration, and its calculation formula is given in Equation (5). If the electrodes are parallel plate electrodes, the capacitance in vacuum *C*_0_ is calculated using Equation (6). Thus, the calculation formula for the relative permittivity of the sample in parallel plate electrodes is given in Equation (7).

(5) $$\varepsilon_{r}=\frac{C_{x}}{C_{0}}$$

(6) $$C_{0}=\frac{\varepsilon_{0}S}{d}$$

(7) $$\varepsilon_{r}=\frac{C_{x}d}{\varepsilon_{0}S}$$

where *d* is the thickness of the sample to be tested, m; *ε*_0_ is the permittivity of free space, F/m; *S* is the electrode area, m^2^.

## 3. Experimental Results and Discussion

### 3.1. Mechanical Property Analysis

**Figure 5:**
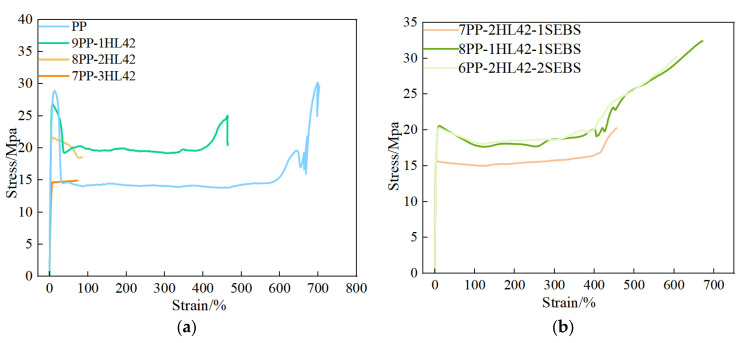
Tensile test result diagram of composite materials. (**a**) Stress–strain curves of PP and PP-HL42; (**b**) Stress–strain curves after adding SEBS.

Figure 5 illustrates the tensile stress-strain responses of the samples, with the curves sequentially displaying four stages: elastic region, yield region, strain softening, and strain hardening. In the elastic region, stress is proportional to strain, accompanied only by reversible changes in bond lengths and bond angles. After yielding, spherulite/lamella sliding occurs, leading to a sharp decrease in the slope of the curve and the formation of a stress plateau. Further stretching induces the orientation and rearrangement of molecular chains, manifesting as strain hardening [30].

**Figure 6:**
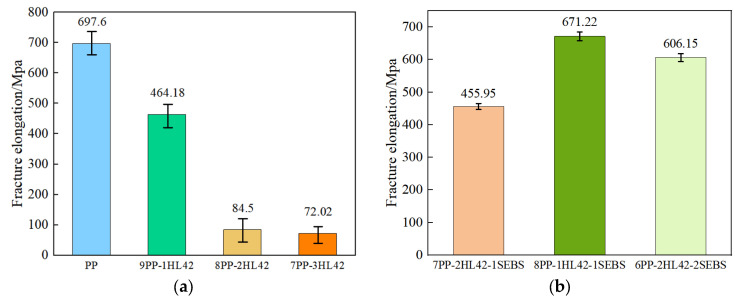
Results of the material’s elongation at break. (**a**) The elongation at break of PP and PP-HL42, (**b**) The elongation at break of the composite with SEBS added.

Figure 6 presents the elongation at break of the samples and the standard deviation of five replicate measurements. As indicated by the standard deviation, the PP and PP/HL42 materials exhibit poor uniformity. However, after the incorporation of SEBS, the error bars shrink significantly, demonstrating improved material homogeneity.For the binary PP/HL42 system (Figure 6a), with the increase in HL42 content, the stress–strain curves gradually become incomplete, and both the breaking stress and elongation at break decrease significantly. When the HL42 filling content reaches 30%, the curve only retains the elastic and yield regions, and the elongation at break plummets from 697.6% (for pure PP) to 72.02%, a reduction of nearly one order of magnitude. Since HL42 has a spherical structure, its relatively low specific surface area limits the contact area with the PP matrix [31]. Moreover, the significant differences in interfacial polarity and chemical structure between the glass wall of HL42 and the plastic (PP) result in extremely poor interfacial bonding and compatibility [32]. This poor interfacial interaction forms stress concentration points within the material, which are prone to crack initiation and propagation under tensile stress, ultimately leading to a substantial deterioration in the tensile properties of the composite materials [33]. To improve the compatibility between PP and HL42 and enhance the tensile properties of the composite materials, toughening modification is necessary. Therefore, SEBS, a thermoplastic elastomer, was selected as the toughening agent. Through its interfacial compatibility and toughening effects, SEBS can alleviate the interfacial bonding issues between PP and HL42, thereby optimizing the mechanical properties of the materials.

The right Figure 5b and Figure 6b shows the tensile stress-strain curves and the elongation-at-break results after the incorporation of SEBS, respectively. Compared with the PP/HL42 composite materials without SEBS, it can be seen that the introduction of SEBS significantly improves the tensile properties of the system. When the filling amount of HL42 is 10% and SEBS is 10%, the tensile stress reaches 32.40 MPa, and the elongation at break is 671.22%, an increase of about 800%. As a toughening agent, SEBS can improve the dispersion uniformity of HL42 in the PP matrix through its own interfacial activity, reducing stress concentration points caused by HL42 agglomeration. It can also effectively regulate the interfacial bonding state between PP and HL42, enhancing the compatibility between the two phases and thereby improving the stress transfer capability of the composite materials under external forces, ultimately achieving optimization of mechanical properties [34,35,36,37]. Since the mechanical properties of the PP/HL42 composite materials are severely insufficient, two ratios, 8PP-1HL42-1SEBS and 6PP-2HL42-2SEBS, were selected for further investigation.

### 3.2. Hardness Results Analysis

**Figure 7:**
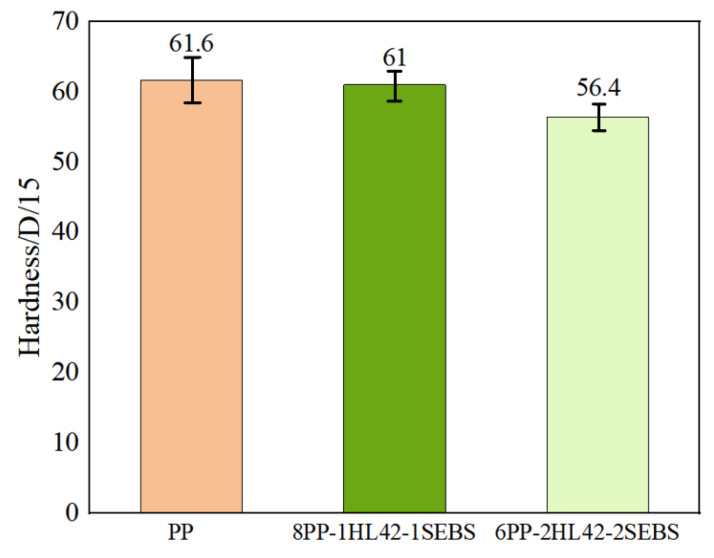
Shore hardness test results.

The Shore hardness test results (Figure 7) indicate that as the contents of HGM and SEBS increase simultaneously, the hardness decreases gradually, while the data dispersion continuously reduces. The reduced dispersion suggests improved system homogeneity and fewer internal defects, which is conducive to enhancing the material consistency. The reduction in hardness implies that the matrix is more likely to undergo shear yielding rather than brittle cracking: under external force, plastic deformation occurs before the nucleation of crazing, thereby increasing the fracture toughness. Meanwhile, the low hardness allows stress concentration areas to be dispersed, significantly reducing the size and density of microcracks [38,39]. Therefore, the PP system toughened by the synergistic effect of HGM and SEBS has achieved better damage tolerance while maintaining lightweighting.

### 3.3. Density Results Analysis

**Figure 8:**
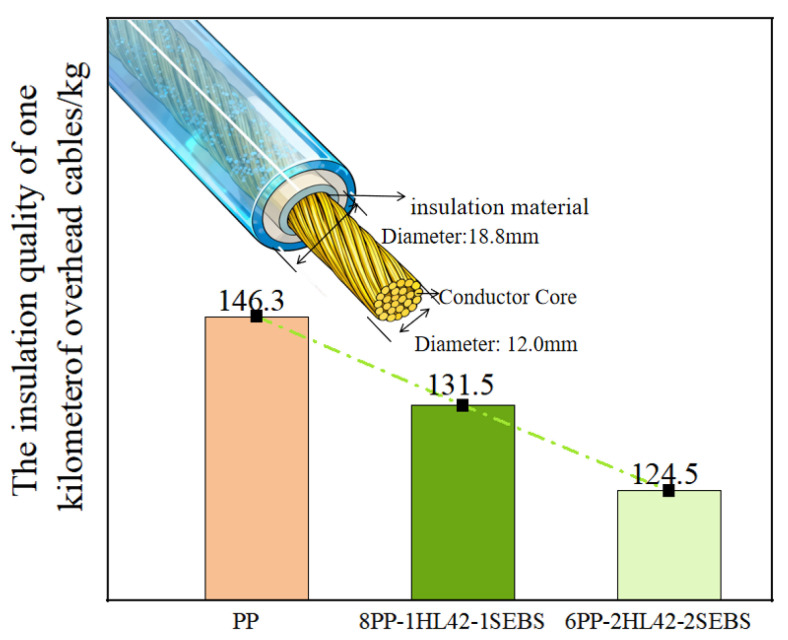
Cable structure diagram and mass of 1 km-long cable with different insulation materials.

From the density test data provided (Table 2), it can be seen that the density of the melt-blended PP-HL42-SEBS composite decreases with the increasing content of HGM. The density of 6PP-2HL42-2SEBS is 15% lower than that of pure PP, indicating a reduction of 133 kg in mass per cubic meter. For overhead cables (Figure 8), the insulation weight of a 1 km-long PP insulated overhead cable (insulation thickness: 3.4 mm; PP density: 0.890 g/cm^3^; conductor nominal cross-sectional area: 120 mm^2^; conductor diameter: 12.0 mm) is approximately 146.3 kg. When the insulation material of the overhead cable with the same dimensions is replaced with 6PP-2HL42-2SEBS, the insulation weight is about 124.5 kg, a reduction of approximately 21.8 kg. This can significantly reduce the load on supports such as utility poles and transmission towers, thereby extending the service life of the overhead cables.

### 3.4. Rheological Property Analysis

**Figure 9:**
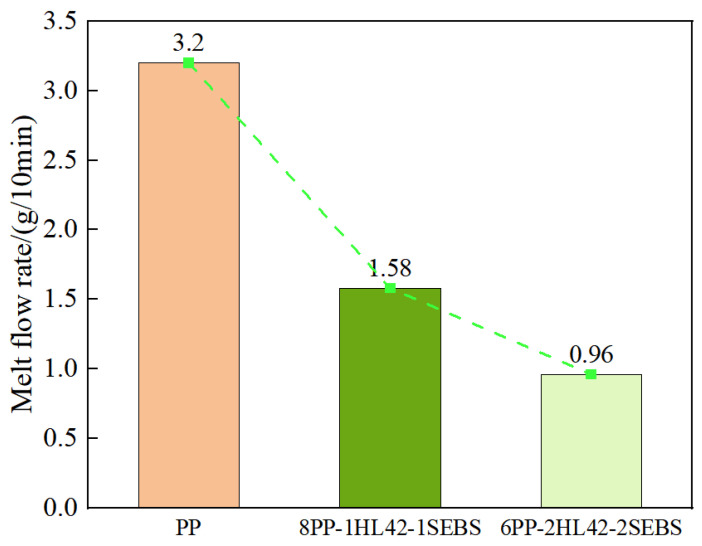
Melt flow index of three materials.

The test results are shown in Figure 9. From the results, it can be seen that the MFR values of PP, 8PP-1HL42-1SEBS, and 6PP-2HL42-2SEBS are 3.20 g/10 min, 1.58 g/10 min, and 0.96 g/10 min, respectively. It is indicated that the addition of HGM and elastomer impairs the fluidity of the composite, indirectly reflecting an increase in material viscosity. With the increase in HL42 and SEBS contents, HGM, as rigid particles, hinders the movement of PP molecular chains. Furthermore, the mutual contact and friction between HGM particles form a skeleton-like structure, which further exacerbates the hindrance to the movement of PP molecular chains. This results in a decrease in the melt flow index (MFI) of the composite and a corresponding increase in viscosity. Consequently, the eccentricity caused by gravity is reduced, leading to more uniform insulation distribution. This not only achieves weight reduction but also better meets the application requirements of overhead cables [40,41].

### 3.5. Electrical Property Analysis

#### 3.5.1. Volume Resistivity Results Analysis

As can be seen from Table 3, the volume resistivities of PP, 8PP-1HL42-1SEBS, and 6PP-2HL42-2SEBS are 8.00 × 10^14^ Ω m, 1.66 × 10^12^ Ω·m, and 1.04 × 10^12^ Ω·m, respectively. By comparing the performance differences between the two SEBS-modified composites, it is found that compared with pure PP, the addition of HGM and elastomer leads to a significant decrease in the volume resistivity of the material, with a reduction of nearly two orders of magnitude, which intuitively reflects the initial impact of these two components on the insulation performance. When the contents of HGM and elastomer are further increased (from 8PP to 1HL42-1SEBS to 6PP-2HL42-2SEBS), the downward trend of volume resistivity slows down significantly, and the numerical change is minimal. This regularity indicates that reasonable regulation of the addition ratio of HGM and elastomer can achieve a more optimal solution between balancing the insulation performance of the material and meeting other modification requirements.

#### 3.5.2. AC Breakdown Results Analysis

**Figure 10:**
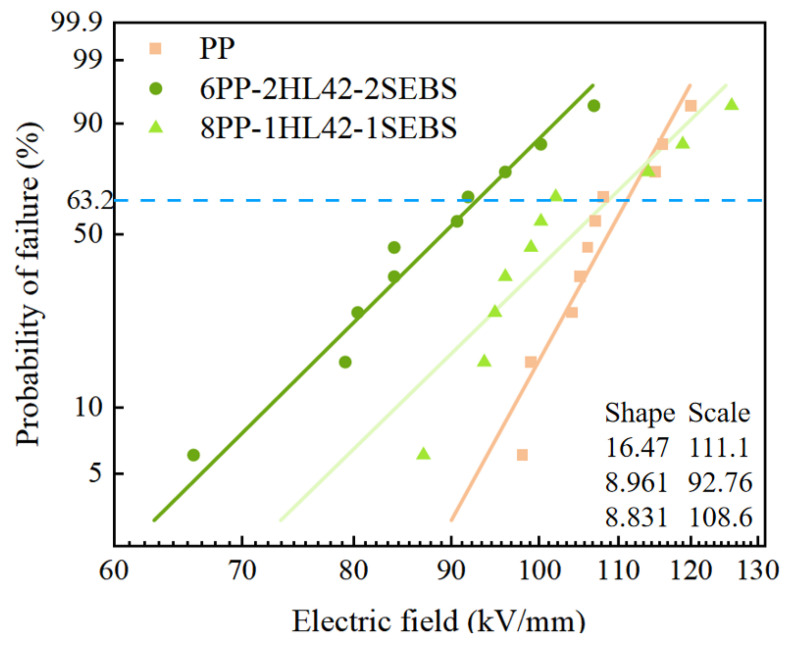
Weibull distribution plot of AC electrical endurance characteristics for three materials.

Figure 10 presents the Weibull probability plots of the breakdown strength data for three materials (PP, 6PP-2HL42-2SEBS, and 8PP-1HL42-1SEBS) analyzed based on the Weibull distribution. Herein, the scale parameter of the Weibull distribution characterizes the characteristic breakdown strength of the material, which refers to the electric field strength corresponding to the breakdown of approximately 63.2% of the samples. As can be observed from the data in the figure, pure PP exhibits the highest characteristic breakdown strength, while the composites incorporated with HGM and SEBS demonstrate differentiated breakdown behaviors: the 6PP-2HL42-2SEBS composite shows the lowest characteristic breakdown strength of 92.76 kV/mm, and the 8PP-1HL42-1SEBS composite has a characteristic breakdown strength of 108.6 kV/mm, which falls between that of pure PP and 6PP-2HL42-2SEBS. The primary reasons for this phenomenon are as follows: a weak interface exists between PP and HGM, which is prone to forming microvoids, thereby inducing electric field distortion and local field strength concentration; simultaneously, the introduction of HGM and SEBS may bring in impurity ions and generate charge traps, ultimately leading to a slight decrease in the breakdown strength of the composites compared to pure PP [42].

#### 3.5.3. Dielectric Loss Results Analysis

As shown in Table 4, the dielectric loss tangent (tanδ) and relative permittivity (ε_r_) of the pure PP matrix are 5 × 10^−5^ and 2.20, respectively, exhibiting the characteristics of low loss and low polarization typical of non-polar semicrystalline polymers. When 10 phr of HL42 and SEBS are added to the PP matrix, the tanδ and ε_r_ of the composite increase to 2.76 × 10^−4^ and 2.24, respectively, with a significant increase in tanδ while only a slight rise in ε_r_. When the addition amount of HL42 and SEBS is increased from 1 phr to 2 phr, the tanδ further increases to 3.92 × 10^−4^, and the ε_r_ rises to 2.30. Further analysis of the data variation trend reveals that with the increase in the addition amount of HL42 and SEBS, the increasing amplitude of tanδ and ε_r_ shows a decreasing trend. This phenomenon indicates that the interfacial polarization effect inside the material is close to saturation, and it can be inferred that if the content of HGM and elastomer is continuously increased subsequently, the deterioration effect on the dielectric parameters of the composite will tend to be limited.

## 4. Mechanism Analysis

**Figure 11:**
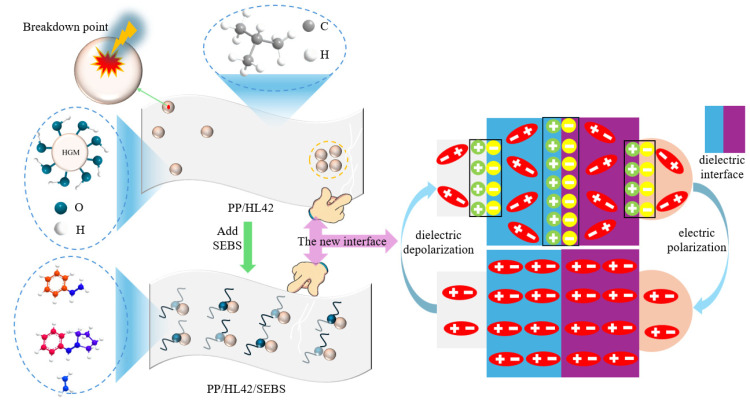
Mechanism analysis of adding HGM and SEBS into PP.

Figure 11 intuitively shows that the glass wall of HL42 is rich in polar hydroxyl groups (-OH), which results in poor interfacial compatibility with the non-polar PP matrix (dominated by -CH_3_ groups) due to significant polarity differences, leading to a reduced contact area and extremely poor interfacial bonding performance. The microspheres are prone to agglomeration and rapid debonding during tensile stress, resulting in a sharp drop in strength and elongation at break [43]. After the introduction of SEBS, its PS end-blocks interpenetrate with PP chains, and its interfacial activity improves the dispersion uniformity of HL42 in the PP matrix, thereby optimizing the mechanical properties of the material [44].

However, toughening does not eliminate the electrical weaknesses at the HGM/PP interface. Due to the poor interfacial compatibility between PP and HGM, a tight bond cannot be formed at the interface, leading to the formation of numerous microvoids, interfacial defects, and discontinuous regions [45]. Under the influence of an external electric field, the presence of the interface causes uneven electric field distribution and significantly enhances interfacial polarization effects. Charges, affected by polarization and electric field gradients, are highly likely to accumulate at interfacial defects, gradually forming local high electric field intensity regions that accelerate the migration and conduction of charges, directly causing a decrease in the overall resistivity of the material. The continuous accumulation of charges at the interface further forms a space charge layer, which significantly exacerbates electric field distortion and induces partial discharge. The energy dissipation during partial discharge directly leads to increased dielectric loss [46]. Meanwhile, the difference in dielectric constants between PP and HGM generates additional polarization effects at the interface. The formation and repeated flipping of dipoles during polarization introduce additional conductive losses and compound the effects of the space charge layer, further exacerbating electric field distortion and ultimately leading to a decrease in breakdown field strength. Moreover, impurities left over from the production of HGM (such as metal ions) and its own internal defects (such as microcracks and pores) become weak points for electric field concentration. These weak points not only induce new partial discharges but also provide pathways for charge migration, ultimately leading to a significant deterioration in the overall dielectric properties of the composite material. Therefore, although SEBS alleviates mechanical degradation, it does not eliminate the interfacial electrical defects introduced by HGM, and lightweight PP insulation materials still need to seek a new balance between compatibility and dielectric properties.

## 5. Conclusions

In this study, polypropylene (PP) was used as the matrix, and hollow glass microspheres (HGM) and SEBS elastomer were incorporated via melt blending. The feasibility of the composite as a lightweight insulating material for low-voltage overhead cables was systematically evaluated. The results indicate that the addition of SEBS significantly improves tensile strength and reduces hardness while maintaining flexibility. The HGM content exhibits a linear negative correlation with density, endowing the material with excellent lightweight properties. In terms of electrical performance, the volume resistivities of the 8PP-1HL42-1SEBS and 6PP-2HL42-2SEBS composites reach 1.66 × 10^12^ Ω·m and 1.04 × 10^12^ Ω·m, respectively, with characteristic breakdown field strengths of 108.6 kV/mm and 92.76 kV/mm, and the dielectric loss remains at a low level. Furthermore, under the condition of 3.4 mm insulation thickness, the weight of the cable can be reduced by approximately 21.8 kg per kilometer, and the weight reduction efficiency increases with the increase in insulation thickness. In conclusion, the PP/HGM/SEBS composite achieves significant weight reduction while meeting the synergistic requirements of electrical and mechanical properties, providing a feasible solution for the lightweight design of low-voltage overhead cables.

## Figures and Tables

**Table 1:** Components of the materials.

Materials	T30s/Phr	HL42/Phr	SEBS/Phr
PP	100	0	0
7PP-3HL42	70	30	0
8PP-2HL42	80	20	0
9PP-1HL42	90	10	0
7PP-2HL42-1SEBS	70	20	10
8PP-1HL42-1SEBS	80	10	10
6PP-2HL42-2SEBS	60	20	20

**Table 2:** Data table for density test.

Materials	Density (g/cm^3^)
PP	0.890
8PP-1HL42-1SEBS	0.801
6PP-2HL42-2SEBS	0.757

**Table 3:** Test results of volume resistivity for three materials.

Materials	Volume Resistivity (Ω·m)
PP	8.00 × 10^14^
6PP-2HL42-2SEBS	1.04 × 10^12^
8PP-1HL42-1SEBS	1.66 × 10^12^

**Table 4:** Dielectric loss (tanδ) and relative permittivity (ε_r_) of PP and composites with different components.

Materials	tanδ (1)	ε_r_ (1)
PP	5 × 10^−5^	2.20
8PP-1HL42-1SEBS	2.76 × 10^−4^	2.24
6PP-2HL42-2SEBS	3.92 × 10^−4^	2.30

## Data Availability

The original contributions presented in this study are included in the article. Further inquiries can be directed to the corresponding authors.

## References

[1] Liao Y., Li R., Shen C., Gong B., Yin F., Wang L. (2022). A Service Life Prediction Method of Stranded Carbon Fiber Composite Core Conductor for Overhead Transmission Lines. Polymers.

[2] Wang J., Li W., Zhang W., Wan B., Zha J. (2024). Aging and life control of cross-linked polyethylene as cable insulation material. Acta Phys. Sin..

[3] Wang X., Wang Y., Ye X., Wu K. (2022). Study on Storage Activity of Cross-Linkable Polyethylene Material Used for High-Voltage Cables. IEEE Trans. Dielectr. Electr. Insul..

[4] Said A.R., Nawar A.G., Elsayed A.E., Abd-Allah M.A., Kamel S. (2021). Enhancing Electrical, Thermal, and Mechanical Properties of HV Cross-Linked Polyethylene Insulation Using Silica Nanofillers. J. Mater. Eng. Perform..

[5] Liu Y., Sun J., Chen S., Sha J., Yang J. (2022). Thermophysical properties of cross-linked polyethylene during thermal aging. Thermochim. Acta.

[6] Selvin M., Shah S., Maria H.J., Thomas S., Tuladhar R., Jacob M. (2024). Review on Recycling of Cross-Linked Polyethylene. Ind. Eng. Chem. Res..

[7] Emdadi K., Gandomkar M., Aranizadeh A., Vahidi B., Mirmozaffari M. (2025). Overview of Monitoring, Diagnostics, Aging Analysis, and Maintenance Strategies in High-Voltage AC/DC XLPE Cable Systems. Sensors.

[8] Dong X., Fan X., Wang W. (2025). Comprehensive Performance Regulation and Characterization of Polypropylene/Elastomer Composite Insulation Materials. Polymers.

[9] Goto T., Ashihara S., Yamazaki T., Okajima I., Sako T., Iwamoto Y., Ishibashi M., Sugeta T. (2011). Continuous Process for Recycling Silane Cross-Linked Polyethylene Using Supercritical Alcohol and Extruders. Ind. Eng. Chem. Res..

[10] Laredo G.C., Reza J., Ruiz E.M. (2023). Hydrothermal liquefaction processes for plastics recycling: A review. Clean. Chem. Eng..

[11] Hosier I.L., Vaughan A.S., Swingler S.G. (2011). An investigation of the potential of polypropylene and its blends for use in recyclable high voltage cable insulation systems. J. Mater. Sci..

[12] Nazrin A., Kuan T.M., Mansour D.A., Faradee R.A., Ariffin A.M., Rahman M.S.A., Abdul Wahabd N.I. (2024). Innovative approaches for augmenting dielectric properties in cross-linked polyethylene (XLPE): A review. Heliyon.

[13] Sui H., Wu K., Zhao G., Yang K., Dong J., Li J. (2024). Greatly enhanced temperature stability of eco-friendly polypropylene for cable insulation by multifold long-chain branched structures. Chem. Eng. J..

[14] He Y., Pan Z., Song H., Ding J., Wang K., Yang J., Zhao X. (2025). Comparison of Production Processes and Performance Between Polypropylene-Insulated and Crosslinked-Polyethylene-Insulated Low-Voltage Cables. Energies.

[15] Huang X., Zhang J., Jiang P., Tanaka T. (2020). Material progress toward recyclable insulation of power cables part 2: Polypropylene-based thermoplastic materials. IEEE Electr. Insul. Mag..

[16] Wang M., Hu S., Zhang W., Zhou Y., Huang S., Zhang J., Zhang Q., Yang C., Li Q., Yuan H. (2024). Electrical properties enhancement of dually grafting modification for polypropylene cable insulation. J. Appl. Polym. Sci..

[17] Gao M., Yang J., Zhao H., He H., Hu M., Xie S. (2019). Preparation Methods of Polypropylene/Nano-Silica/Styrene-Ethylene-Butylene-Styrene Composite and Its Effect on Electrical Properties. Polymers.

[18] Nam C.Y., Lee J.H., Kim M.A., Yoon H.G. (2025). High Performance and Recyclable Polypropylene/Styrene-Ethylene-Butylene-Styrene Blends for Next Generation Cable Insulation with Enhanced Breakdown Strength Through Controlling Crystallinity. Polymers.

[19] Niu S., Zhang T., Zhang H., Zhang C., Zhang Y., Yin C., Zhang Y., Wu G., Chi Q. (2025). Balancing the Mechanical Toughness and Electrical Insulation of Polypropylene by Blending and Grafting Modifications. Macromol. Chem. Phys..

[20] Hanif M.A., Shin H., Chun D., Kim H.G., Kwac L.K., Han S.-W., Kang S.-S., Kim Y.S. (2024). Development of Highly Ultraviolet-Protective Polypropylene/TiO_2_ Nonwoven Fiber. J. Compos. Sci..

[21] An Z., Zhang J., Pan S. (2009). Low-density core-shell composite hollow microspheres with tunable magnetic properties. J. Phys. Chem. Solids.

[22] Qiao Y., Li Q., Li Q., Bian X., Lu C., Yang K., Zheng T., Zhang X., Wang X. (2022). Lightweight epoxy foams prepared with arranged hollow-glass-microspheres/epoxy hollow spheres. Compos. Commun..

[23] Zhu Z., Wang J., Liu Y., Xian G., Wang Y., Wu C., Peng X., Fang Y., Kong L. (2022). Effect of Hollow Glass Microspheres with Different Contents and Types on Properties of Polypropylene Composites. ChemistrySelect.

[24] Galvagnini F., Dorigato A., Fambri L., Pegoretti A. (2022). Development of Novel Polypropylene Syntactic Foams Containing Paraffin Microcapsules for Thermal Energy Storage Applications. Molecules.

[25] Ren H., He Z., Li D., Zhang L., Chen L., Lou Y., Xu M. (2019). Synergistic enhanced yield strength, tensile ductility and impact toughness of polydicyclopentadiene nanocomposites by introducing low loadings of di-functionalized silica. Polym. Test..

[26] Baptista C.A., Canevarolo S.V. (2019). Grafting polypropylene over hollow glass microspheres by reactive extrusion. Polímeros.

[27] (2018). Plastics-Determination of Tensile Properties-Part 3: Test Conditions for Films and Sheets.

[28] (2011). Plastics-Determination of the Melt Mass-Flow Rate (MFR) and Melt Volume-Flow Rate (MVR) of Thermoplastics-Part 1: Standard Method.

[29] (2013). Electrical Strength of Insulating Materials-Test Methods-Part 1: Tests at Power Frequencies.

[30] Ma H., Li T., Pan B., Li J., Jiang S., Peng X., Jing L. (2022). Tensile behaviour of isotactic polypropylene with different crystallinities and service temperatures. Polym. Test..

[31] Leidner J., Woodhams R.T. (1974). The strength of polymeric composites containing spherical fillers. J. Appl. Polym. Sci..

[32] Zhang C., Dai G. (2006). Effects of Interface Coupling Reaction on the Rheology of Polypropylene/Glass Bead System. J. East China Univ. Sci. Technol..

[33] Liang J., Li R. (2004). Mechanical properties and morphology of glass bead–filled polypropylene composites. Polym. Compos..

[34] Ma Y., Du Y., Zhao J., Yuan X., Hou X. (2020). Preparation and Characterization of Furan–Matrix Composites Blended with Modified Hollow Glass Microsphere. Polymers.

[35] Yan J., Wang C., Zhang T., Xiao Z., Xie X. (2024). Super Tough PA6/PP/ABS/SEBS Blends Compatibilized by a Combination of Multi-Phase Compatibilizers. Materials.

[36] Afolabi O.A., Kanny K., Mohan T.P. (2022). Analysis of Particle Variation Effect on Flexural Properties of Hollow Glass Microsphere Filled Epoxy Matrix Syntactic Foam Composites. Polymers.

[37] Dorigato A., Fredis G. (2024). Effect of nanofillers addition on the compatibilization of polymer blends. Adv. Ind. Eng. Polym. Res..

[38] Galvagnini F., Dorigato A., Fambri L., Fredi G., Pegoretti A. (2021). Thermophysical Properties of Multifunctional Syntactic Foams Containing Phase Change Microcapsules for Thermal Energy Storage. Polymers.

[39] Yee A.F., Pearson R.A. (1986). Toughening mechanisms in elastomer-modified epoxies. J. Mater. Sci..

[40] Liang J., Chen C., Zou S., Tsui C., Tang C., Zhang S. (2015). Melt flow behavior of polypropylene composites filled with multi-walled carbon nanotubes during extrusion. Polym. Test..

[41] Li X., Chen X., Liu L., Wei Y., Hao C., Li S., Li G. (2023). The influence and mechanism of elastomer types on electrical and mechanical properties of polypropylene insulation materials for high voltage cables. J. Appl. Polym. Sci..

[42] Wang P., Zhong S., Yan K., Liao B., Guo Y., Zhang J. (2024). Effect of hollow glass microspheres surface modification on the compressive strength of syntactic foams. J. Mater. Res. Technol. JMRT.

[43] Bîrleanu E., Mihăilă I., Topală I., Borcia C., Borcia G. (2023). Adhesion Properties and Stability of Non-Polar Polymers Treated by Air Atmospheric-Pressure Plasma. Polymers.

[44] Xing Z., Gu Z., Zhang C., Guo S., Cui H., Lei Q., Li G. (2022). Influence of Space Charge on Dielectric Property and Breakdown Strength of Polypropylene Dielectrics under Strong Electric Field. Energies.

[45] Li G., Gu Z., Xing Z., Zhang C., Guo S., Hao C., Lei Q. (2022). Space Charge and Trap Distributions and Charge Dynamic Migration Characteristics in Polypropylene under Strong Electric Field. ECS J. Solid State Sci. Technol..

[46] Zhang L., Zhang Y., Zhou Y., Teng C., Peng Z., Spinella S. (2018). Crystalline Modification and Its Effects on Dielectric Breakdown Strength and Space Charge Behavior in Isotactic Polypropylene. Polymers.

